# Pyeloureteral Anastomosis as a Reconstructive Technique for Post-Renal Transplant Ureteral Stenosis

**DOI:** 10.5152/tud.2023.23105

**Published:** 2023-11-01

**Authors:** Irene de la Parra, Juan Gómez Rivas, Beatriz Gutíerrez, Sarelis Infante, Isabel Galindo, Marco Ciappara, Jesús Blázquez, Ángel Gómez, Jesús Moreno-SIerra

**Affiliations:** Clinico San Carlos Hospital, Madrid, Spain

**Keywords:** Renal transplant, ureteral stenosis, open surgery, pyeloureteral anastomosis, reconstructive surgery

## Abstract

**Objective:**

Ureteral stenosis in renal transplant patients is a frequent urological complication that involves significant morbidity and may compromise graft function. Despite the common use of minimally invasive techniques, surgery continues to be the definitive treatment for ureteral stenosis, and pyeloureteral anastomosis is an infrequent but effective technique in the management of this pathology and has been described as a safe treatment with a low percentage of complications.

**Methods:**

This is a retrospective study of patients in whom surgical intervention via pyeloureteral anastomosis was carried out in our center in the last 12 years. A descriptive analysis of perioperative management, complications, and functional results is provided. A comparison of renal function at diagnosis and after surgery was made to evaluate the effectiveness of the procedure.

**Results:**

Thirteen patients underwent surgery within the described time frame. Time to diagnosis of stenosis was 60 days [interquartile range (IQR) 31-368]. Creatinine at diagnosis was 2.2 mg/dL [IQR 1.9-3] with a glomerular filtration rate, estimated by the modification of diet in renal disease equation, of 29 mL/min/1.73 m^2^ [IQR 22.6-34.5]. Of these patients, 92.3% underwent percutaneous nephrostomy, and 38.5% also had a ureteral catheter. The mean duration of surgery was 265 minutes [IQR 240-300], and hospital stay was 9 days [IQR 7.5-16]. A double J was placed in all cases, which was maintained for 36 days [IQR 30-49]. Postoperative complications occurred in 15.4% of patients. Serum creatinine 1 year after surgery was 1.6 ± 0.4 mg/dL. Among the patients, 76.9% had no new pyelocalyceal dilatation on follow-up Doppler ultrasound scans at a mean follow-up time of 12 months. The restenosis rate was 23.1%, and all were successfully treated by endoscopic approach. There was an improvement in renal function figures at 1, 3, 6, and 12 months compared to renal function at diagnosis, both in terms of serum creatinine and glomerular filtration rate, with statistically significant results.

**Conclusion:**

Pyeloureteral anastomosis as a reconstructive technique of the urinary tract in renal transplant patients is an effective and reproducible technique with good long-term results.

**Main Points**

- Ureteral stenosis after renal transplantation is a frequent complica- tion and can compromise the renal function of the graft.
- The most frequent presentation of a ureteral stenosis is the finding of pyelocalyceal and ureteral dilatation with deterioration of renal function.
- Minimally invasive techniques, such as balloon dilatation, are frequently used, but their success rate is limited.
- Pyeloureteral anastomosis has proven to be effective and safe in the management of patients with ureteral stenosis after renal transplantation.

## Introduction

Urinary tract complications are a significant cause of morbidity after renal transplantation,^1-3^ varying in incidence from 3% to 4%. About 10-15% of patients with urinary tract complications have secondary graft function impairment, and a mortality rate of up to 15%.^3^ Ureteral stenosis is one of the most frequent complications, with an incidence of 0.6%-10.5%.^4^ Unfortunately, not much has been published on the topic, with most of the publications being about the description of the technique. The most frequent presentation is the finding of pyelocalyceal and ureteral dilatation with deterioration of renal function. In addition, there may be a possible associated infectious complication, with urinary diversion being the initial management,^5^ and later performing a complete study of the urinary tract and propose a definitive solution. Minimally invasive techniques, such as balloon dilatation, are frequently used; however, their success rate is limited (45%-62%),^6,7^ making definitive surgical techniques necessary. The use of the ipsilateral native approach is one of them, and although its use is widely accepted, there are not many reviews in the literature regarding its implications and functional results. The present article reviews the existing literature and presents our experience in open pyeloureterostomies during the last 12 years. We believe that our experience can help to promote the use of this safe technique with satisfactory results.

## Material and Methods

We conducted a retrospective observational study of all renal transplants performed at our center from January 2010 to December 2021, identifying patients with ureteral stenosis after transplantation and those who were treated by pyeloureteral anastomosis, either primary or iteratively. The Ethical Committee of Clinico San Carlos Hospital approved the collection of the data for this study, with the intern code 23-038 on the act 7.1/2023. Informed consent was obtained previously from each of these patients.

After reviewing the existing literature, we performed a descriptive scrutiny, analyzing the characteristics, surgical technique, and functional results, and a comparative analysis of renal function as a parameter of success of the technique.

### Variables to Be Studied

Patients undergoing renal replacement therapy or predialysis who underwent renal transplantation were selected for analysis. The following demographic variables were collected at the time of transplantation: sex, age, comorbidity, type of donation, graft laterality, cold ischemia time, surgical technique of ureterovesical anastomosis, and double J catheter time. Regarding the diagnosis of stenosis, we recorded the main sign or symptoms and method of diagnosis, mean time to stenosis, renal function, and the need or not for urgent surgical treatment. On the surgical technique and the reason for our study, we collected the surgical time, hospital stay time, double J time, and the existence or not of postoperative complications. We defined a follow-up of at least 1 year, analyzing renal function at 1, 3, 6 months and 1 year, as well as the resolution of pyelocaliceal dilatation in the control imaging tests.

### Ureteral Stenosis Treatment

In our center, the treatment of ureteral strictures differs according to degree, symptoms, and complexity. Thus, patients with mild symptoms or asymptomatic stenoses are followed by regular renal function analysis and ultrasound/Doppler, whereas those symptomatic or with impaired renal function but short (<1 cm) or moderate and distal at diagnosis are treated primarily by endoscopic approach with balloon dilatation or laser ureterotomy. Finally, open surgical treatment by ureterovesical reimplantation or ureteropielic anastomosis is relegated to cases of long (>1 cm), proximal or multiple strictures when the location of the stricture allows it. 

### Surgical Technique

**Figure 1.**
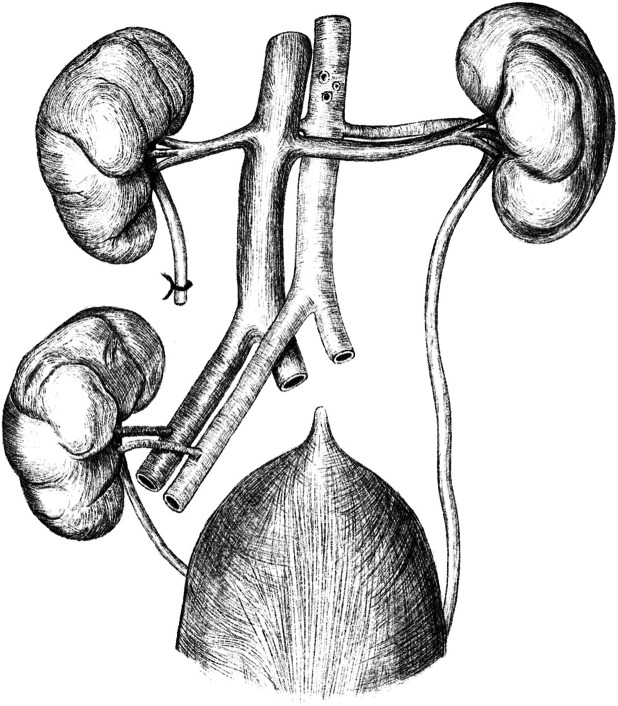
Ureteropelvic anastomosis with ligation of the proximal ipsilateral native ureter.

**Figure 2.**
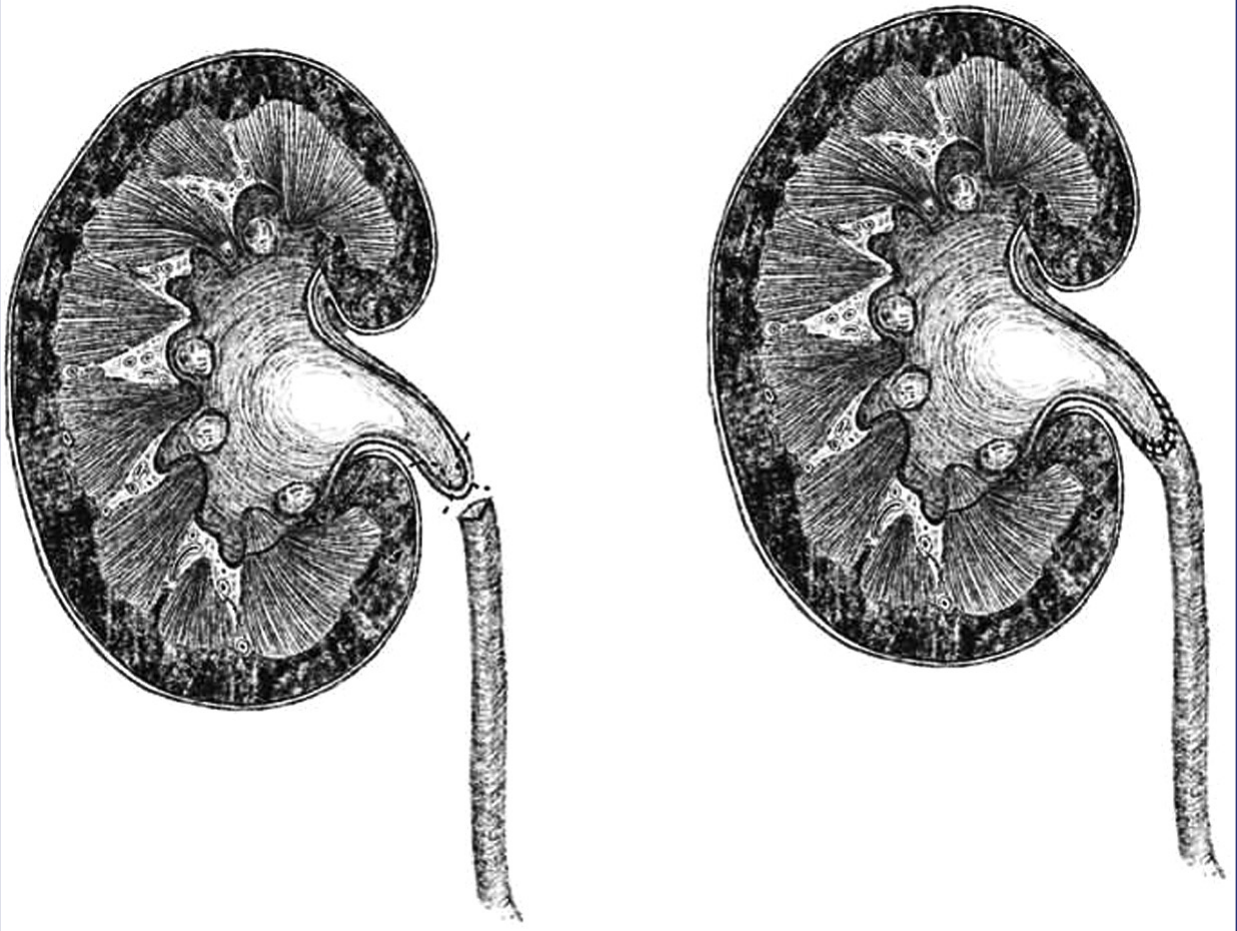
Terminal–terminal ureteropelvic anastomosis.

The first step of surgery is the placement of a double J in the ipsilateral native ureter. A previous retrograde pyelography may be performed to check the indemnity of the native ureter to be used. The approach is performed by Gibson incision, with prior ilioinguinal retroperitoneal access. The native ureter is located, mobilized, and sectioned at the proximal level, and the proximal end is ligated. Subsequently, the renal pelvis of the graft is carefully dissected, avoiding damage to the vascular structures, with sectioning of the latter, and the ureteropelvic anastomosis is performed, either terminoterminal or terminolateral, depending on the surgeon’s preference. For this, a PDS 5-0 suture is used by means of loose stitches over a double J catheter previously placed from the native ureter to the renal pelvis of the graft. In Figures 1 and 2, we can see a basic and illustrative scheme of the surgical technique. One of the principles of reconstructive surgery is to mobilize the 2 healthy ends in order to proceed to perform a tension-free anastomosis. If there is tension, there may be recurrence, so obtaining a tension-free anastomosis is a critical point of the surgery.^8^

### Objectives

This study aimed to evaluate the efficacy of open pyeloureterostomy as a reconstructive technique for ureteral stricture in renal transplant patients, by measuring renal function, and to confirm that it is safe in terms of postoperative complications and survival. We compared our results to those described by the existing series and according to the evidence reviewed.

### Statistical Analysis

The qualitative variables are summarized with their frequency distribution, presenting the quantitative variables normally distributed with mean (standard deviation) and those not normally distributed with median (interquartile range (IQR)). For the comparative analysis of renal function at different times, we used the Friedman’s nonparametric test, considering the small sample size, to analyze whether there are differences in serum creatinine and estimated glomerular filtration rate (GFR) at least at 2 follow-up visits. The significance level was defined as .05. Corrections for multiple comparisons were made using the Bonferroni correction. We used IBM Statistical Package for the Social Sciences Statistics version 26.0 (IBM SPSS Corp.; Armonk, NY, USA) for statistical analyses.

## Results

From January 2010 to December 2021, we performed in the renal transplantation unit of our center 668 renal transplants, identifying 34 patients with ureteral stenosis as a complication. Of these, 13 patients were operated using an open pyeloureteral anastomosis approach, by 2 experienced surgeons. The mean age at renal transplantation was 61 ± 11.5 years, and 11 (84.6%) were male. The Charlson index at the time of transplantation was 5 ± 2.1. The most frequent cause of chronic kidney disease was nephroangiosclerosis, where 6 (46.2%) and 8 (61.5%) were previously on hemodialysis. The mean residual diuresis was 300 cc [IQR 100-1250]. Of them, 10 (76.9%) had received a transplant from a braindead donor, and the mean cold ischemia time was 19 ± 3.9 hours. All patients underwent extravesical ureteroneocystostomy according to the Lich–Gregoir technique during transplantation and maintained the double J for 15 days [IQR 14-30].

The time for ureteral stricture was 60 days [IQR 31-368], with renal function impairment being the most frequent sign at diagnosis (61.5%), while pyelocaliceal dilatation of the graft was the main sign in 5 (38.5%). The mean stenosis diameter was 2.3 ± 0.9 cm, 8 (61.5%) of which were proximal. The serum creatinine at diagnosis was 2.2 mg/dL [IQR 1.9-3] with a GFR of 29 mL/min/1.73 m^2^ [IQR 22.6-34.5]. Twelve (92.3%) had a percutaneous nephrostomy placed in the graft at diagnosis, and 5 (38.5%) also had an antegrade double-J. The average time from the diagnosis to the intervention was 6 ± 2.7 months. The procedure was iterative in 4 (30.8%) of the cases having previously undergone an endoscopic procedure; using balloon dilatation in 3 (23.1%), and open ureteral reimplantation in 1 (7.7%) of the cases.

The median operative time was 265 minutes [IQR 240-300] and hospital stay was 9 days [IQR 7.5-16]. A double J was placed in all cases and maintained for 36 days [IQR 30-49]. Nephrectomy of the ipsilateral native kidney was performed in only 1 (7.7%) case, the first case in the series. One (7.7%) patient required transfusion of 2 units of red blood cell concentrates during surgery. Five (53.9%) patients presented immediate complications: 4 (30.8%) Clavien I and 1 (7.7%) Clavien II: consisting of paralytic ileus in 2 (15.4%); postoperative hematoma in 1 (7.7%) and 1 surgical wound infection (7.7%). In 9 (69.2%) of the patients, there was pyelocaliceal dilatation in the control ultrasound scans, none of them requiring treatment during the follow-up period. Three patients (23.1%) presented symptomatic pyelocaliceal dilatation with renal function impairment and required endoscopic treatment for restenosis. Serum creatinine (mg/dL) was 1.3 [IQR 1.1-1.7], 1.4 [IQR 1.1-1.7], 1.5 [IQR 1.2-1.8], and 1.5 [IQR 1.3-1.8] at 1, 3, 6, and 12 months after surgery, respectively, while GFR (mL/min/1.73 m^2^) was 47 [IQR 38-61.1], 47 [IQR 36.9-61.5], 45 [IQR 38.7-58.5], and 44 [IQR 35.2-55.8], respectively. Table 1 shows the 13 patients with their most representative variables.

Regarding the comparative analysis of renal function, it was found that there were statistically significant differences (*P* < .01) between serum creatinine and GFR at the time of diagnosis of stenosis and at 1, 3, 6, and 12 months, respectively.

**Figure 3.**
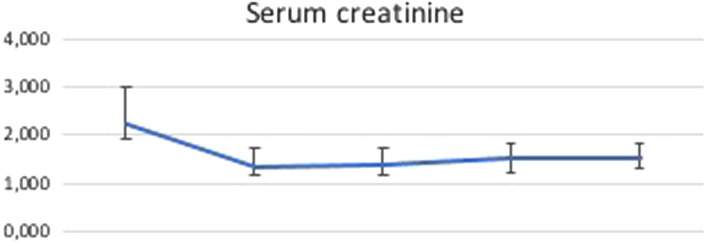
Evolution of serum creatinine levels (median and interquartile range) at diagnosis, after a month, after 3 months, and after a year.

**Figure 4.**
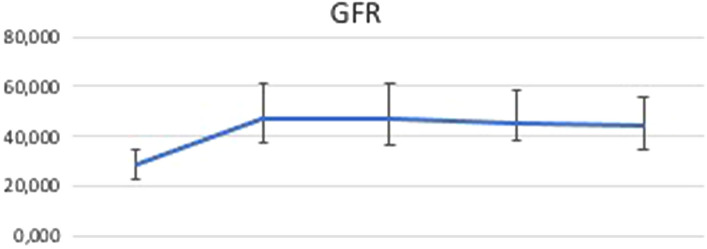
Evolution of glomerular filtration levels (median and interquartile range) at diagnosis, after a month, after 3 months, and after a year. GFR, glomerular filtration rate.

Figures 3 and 4 show the evolution of creatinine levels and GFR at different times during the follow-up period. After the comparative analysis using the Friedman’s test, there were statistically significant differences with a *P*-value of .0001 in both the analysis using creatinine and the estimation of GFR.

## Discussion

In the last 12 years, we have performed 13 open pyeloureterostomies in our center as a reconstructive technique for ureteral stricture after renal transplantation. We recorded a mean hospital stay of 9 days, a postoperative complication rate of 38.4%, and a restenosis rate of 23.1%.

Renal transplantation has become the standard treatment for chronic renal failure and surgical technique is practically the same as that described in 1960. However, reconstruction of the urinary tract during renal transplantation is possible by multiple procedures.^9^ This choice is important since most urological complications involve the ureterovesical anastomosis.^10^ Although the use of the native ureter has been described as a valid technique,^11,12^ ureteroneocystostomy is the most widely accepted technique for reconstruction of the urinary tract during transplantation.^13^ While both techniques show the same risk of urological complications, most surgeons initially opt for Lich–Gregoir ureteroneocystostomy,^14^ reserving pyeloureterostomy as a salvage option in cases of ureterovesical anastomosis complications.^15^ Promeyrat et al^16^ conducted a retrospective study in which they compared the 2 techniques and their different urological complications and concluded that there is no evidence on the superiority of 1 technique over the other, with anuria, recipient gender, and donor age being independent risk factors in the occurrence of complications and the double J placement being a protective factor.

Urological complications [ureteral stricture, vesicoureteral reflux (VUR) and urinary leakage] are the major morbidity cause after renal transplantation.^17^ While ureteral stricture varies from 0.6% to 10.5%,^4^ ureteral necrosis and urinary leakage occur in 1%-5% and the incidence of VUR is as high as 50%, with 0.1%-1.1% of them being severe.^18^ In our series, 34 patients with ureteral stricture (5%), 2 patients with urinary leakage (0.29%), and a single patient with symptomatic VUR (0.15%) were observed, although the number of patients with mild VUR is expected to be higher.

Most ureteral stenoses appear within 90 days and cases have been described even 10 years or more after transplantation.^10^ Its presentation is variable, the most frequent being deterioration of renal function and oligoanuria. Likewise, pyelocaliceal and ureteral dilatation of the graft is observed, with antegrade pyelography after nephrostomy placement being the most effective test to characterize stenosis. In our center, the mean time to onset of stenosis was 60 days [31-368] and deterioration of renal function was the most frequent sign at diagnosis (61.5%). Most of them (92.3%) underwent percutaneous nephrostomy of the graft at diagnosis in order to subsequently define the characteristics of the stenosis.

To perform an open surgical approach, it is important to have a correct knowledge of the characteristics of the stenosis and for this purpose a descending pyelography is essential.^5^ Our group also advocates the performance of a late-phase contrast-enhanced computed tomography scan, if the patient’s renal function allows it, especially to evaluate extrinsic compressive causes.

In 2013, He et al^19^ advocated the importance of classifying ureteral strictures into grades to make treatment decisions. They define grade 1 as impaired renal function with hydronephrosis but without stenosis objectified on pyelography. Grade 2 adds the finding of a distal ureteral stricture < 1 cm. Finally, in grade 3, there is a distal stenosis <1 cm, extending to the proximal or pelvis. As for treatment, they advocate using surgical reconstructive techniques as the best option for grade 3 strictures. According to European clinical guidelines,^20^ the treatment of choice for those stenoses smaller than 3 cm is endoscopic, with a success rate of 50%, although the maximum success is obtained for those <1 cm. In case of recurrence after a primary endourological approach and/or stenosis of more than 3 cm in length, surgical reconstruction, including direct ureteral reimplantation or use of the native ureter, should be performed.

The decision to perform this technique primarily in most of the cases in our series was based on the characteristics of the stenosis, choosing this technique in middle-third or distal stenoses greater than 1 cm and in all proximal stenoses. An important factor for its implementation over the years has been the good functional results previously observed and the experience acquired by the 2 surgeons in its performance.

At least 2 techniques for pyeloureteral anastomosis have been described, terminolateral and terminoterminal, with Leadbetter et al first describing terminal–terminal anastomosis in 1966.^21^ Terminolateral anastomosis has also been described by means of a longitudinal incision of the native ureter and its subsequent anastomosis to the ureterotomy in the graft ureter, as a way of avoiding ligation of the proximal ureteral end.^22^ Table 2 shows the most relevant manuscripts on both these techniques over the years. All of them except 1 are retrospective studies in which they analyze functional performance and success rate. Most of them (6) describe the terminoterminal technique, despite the fact that an important number of studies analyze the terminolateral technique (5), and only in 2 of them the lateroterminal technique was performed. Unfortunately, there are no comparative studies between the techniques, and this reflects the importance of the surgeon’s preference and experience.

Regarding complications, we highlight the fact whether to perform nephrectomy of the ipsilateral native kidney, discussed in the literature, due to the possible infectious complications that ligation of the proximal ureter can trigger. While several authors have described the need to perform nephrectomy in the same surgical procedure,^23,24^ in recent years there has been a trend to be conservative. Riedijer et al^17^ advocate leaving the native kidney in place, since it will finish functioning with the functioning of the graft. However, they advocate performing a renogram in case of a relevant amount of residual diuresis prior to transplantation.

Recently, Hernani M Neto et al^15^ performed a retrospective study of 4215 transplants who underwent pyeloureterostomy without associated native kidney nephrectomy. Their analysis revealed a low incidence (2%) of complications of the native kidney, only requiring nephrectomy of the native kidney in 495 patients. In addition, they point out previous augmentation cystoplasty and adult polycystic disease as risk factors for presenting this condition. In our center, nephrectomy of the native kidney was only performed in 1 case. Its initial performance was indicated due to the surgeon’s preference in relation to the studies performed up to that time, that is, in the year 2010, which advocated its performance. Even though the median residual diuresis in our series was 300 mL and 69.2% of the patients presented pyelocaliceal dilatation of the native kidney in the control ultrasound scans, none presented complications at this level.

As for follow-up, an exhaustive measurement of renal function was performed monthly, and control Doppler ultrasound scans were done every 3 months. As described in the series reviewed, it was shown to be an effective and safe technique, with a low incidence of complications (only 38.4% of complications, all Clavien I or II) and a low rate of restenosis (23.1%).

The limitations of our study include the small sample of patients, the fact that it was only a retrospective, single-center study, and that the selection of the technique depended on the surgeon. We have therefore reviewed the literature and compared it with our results. We emphasize that our series only analyzes patients who undergo this technique as a treatment for ureteral stenosis, while most of the previously published studies have included transplant patients with urinary leakage or VUR as complications, thus adding evidence but also adding heterogeneity. We believe it would be extremely interesting in future to promote studies that analyze the differences in the functional results of this technique for the different urinary complications in renal transplant patients, as well as a comparison between the variations of the technique. For this, it is important to have a larger number of patients, so multicenter studies could provide greater knowledge.

Ureteral stenosis after renal transplantation is a frequent complication and can compromise the renal function of the graft. Although minimally invasive treatments have been described and widely used, in many instances, a definitive treatment is necessary, such as pyeloureterostomy. Despite being an infrequent technique, it has proven to be effective and safe in the management of these patients, with an acceptable success rate. It will be necessary to study larger and multicenter series to be able to extrapolate our results.

## Figures and Tables

**Table 1.** Preoperative, Perioperative, and Postoperative Characteristics of Patients Who Underwent Open Pyeloureterostomy

Patient	Cause of CKD	Substitutive Therapy	Residual Diuresis (mL)	Time to Stenosis (Months)	Previous Actions	Operative Time (Minutes)	Hospital Stay (Days)	Serum Creatinine at Diagnosis (mg/dL)	Serum Creatinine at 1 Year (mg/dL)
1	Nephroangiosclerosis	HD	200	2	PCN, ureteral catheter	240	13	3	1.8
2	Diabetes	HD	50	34	PCN	300	17	3	1.8
3	Hepatorenal polycystic kidney disease	PD	1500	1	PCN	300	15	2	1.3
4	Nephronophthisis	HD	1000	1	PCN	350	9	1.4	1.4
5	Diabetes	PD	1500	5	PCN	400	20	2	1.6
6	Hepatorenal polycystic kidney disease	PD	1500	19	PCN, ureteral catheter	240	5	2.2	1.3
7	Nephroangiosclerosis	HD	100	3	PCN	270	9	1.6	2
8	Nephroangiosclerosis	PD	1000	29	PCN, ureteral catheter	240	8	2.6	1.7
9	Nephroangiosclerosis	HD	100	1	PCN, ureteral catheter	240	20	6.7	2
10	Nephroangiosclerosis	HD	300	4	PCN	240	10	2.1	1.3
11	Nephroangiosclerosis	PD	200	2	PCN	180	7	2.8	1.3
12	Hepatorenal polycystic kidney disease	HD	500	1	PCN, ureteral catheter	300	8	2.8	1.1
13	Unaffiliated	HD	100	4	Conservative	240	6	1.8	1.5

CKD, chronic kidney disease; HD, hemodialysis; PD: peritoneal, dyalisis; PCN, percutaneous nephrostomy.

**Table 2.** Previous Literature About Pyeloureteral Anastomosis

Author	Indication	Sample	Study	Year	Objectives	Method of Anastomosis
Schiff JR et al^25^	Urinary fistulae, ureteral necrosis	N = 7	Retrospective	1981	Success rate	Terminoterminal
Anderson et al^26^	Ureteral stenosis/VUR	N = 4	Retrospective	1982	Results without ligation of the native ureter	Lateroterminal
Baquero et al^27^	Ureterovesical anastomosis avulsion, urinary leakage, ureteral stenosis	N = 7	Retrospective	1985	Results by ligating the native ureter	Terminoterminal
Lord et al^28^	During transplantation and after urological complication	N = 23	Retrospective	1990	Success rate	Terminoterminal
Kockelbergh et al^21^	Urological complications after transplant	N = 5	Clinic cases	1993	Results without ligation of the native ureter	Lateroterminal
Salomon et al^10^	Ureteral stenosis, urinary leakage, VUR	N = 19	Retrospective	1999	Success rate	Terminolateral
Schult et al^9^	Ureteral necrosis, ureteral stenosis	N = 48	Retrospective	2000	Analysis of complications	Terminolateral
Sandhu et al^29^	Ureteral stenosis, VUF	N = 10	Retrospective	2011	Analysis of complications	Terminoterminal
Lehmann K et al^30^	Ureteral stenosis, urinary leakage, VUR	N = 35	Retrospective	2011	Analysis of complications	Termino-lateral
Riediger C et al^17^	Ureteral stenosis, urinary leakage, VUR, ureteral necrosis	N = 16	Retrospective	2014	Success rate	Terminolateral
Trilla E et al^5^	Ureteral stenosis	N = 7	Retrospective	2014	Success rate	Terminolateral
Promeyrat et al^16^	During transplantation	N = 343	Retrospective	2016	Comparison of ureterovesical anastomosis techniques	Terminoterminal
Neto HM et al^15^	During transplantation and after urological complication	N = 495	Retrospective	2021	Results by ligating the native ureter	Terminoterminal

VUF: vesicoureteral fistula; VUR: vesicoureteral reflux.
